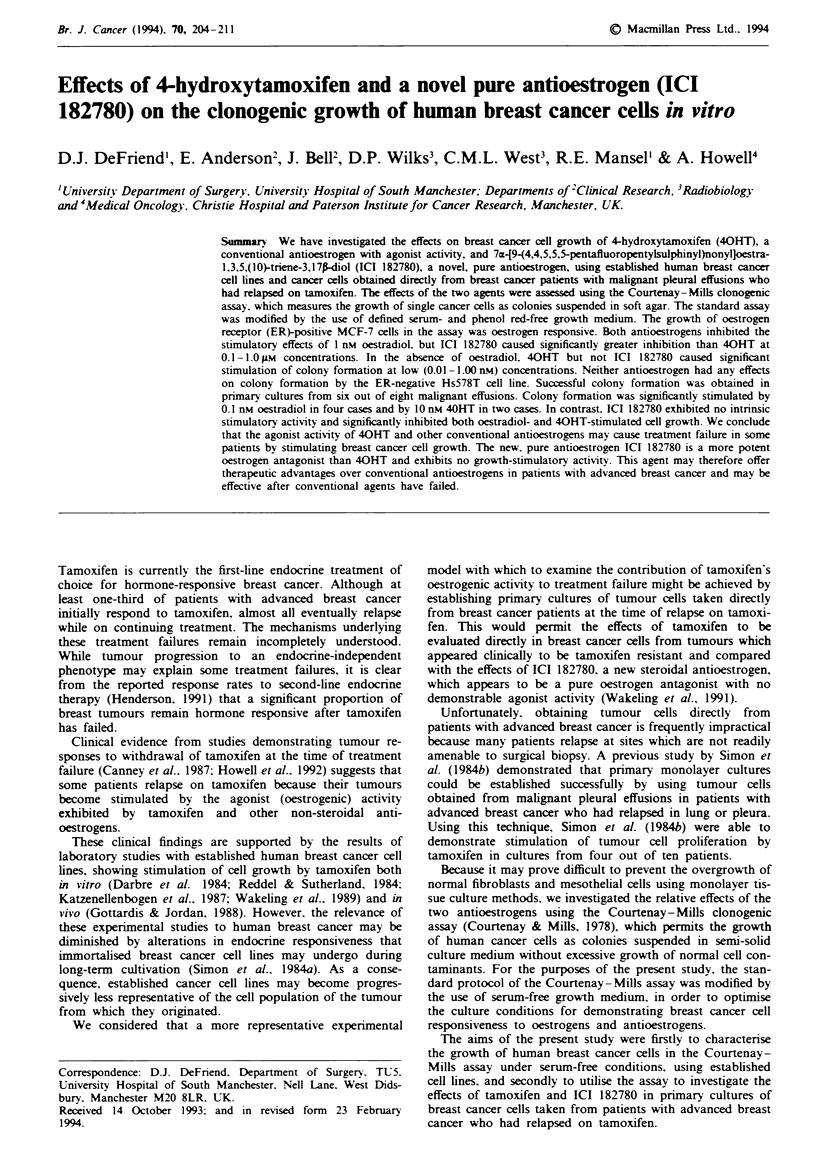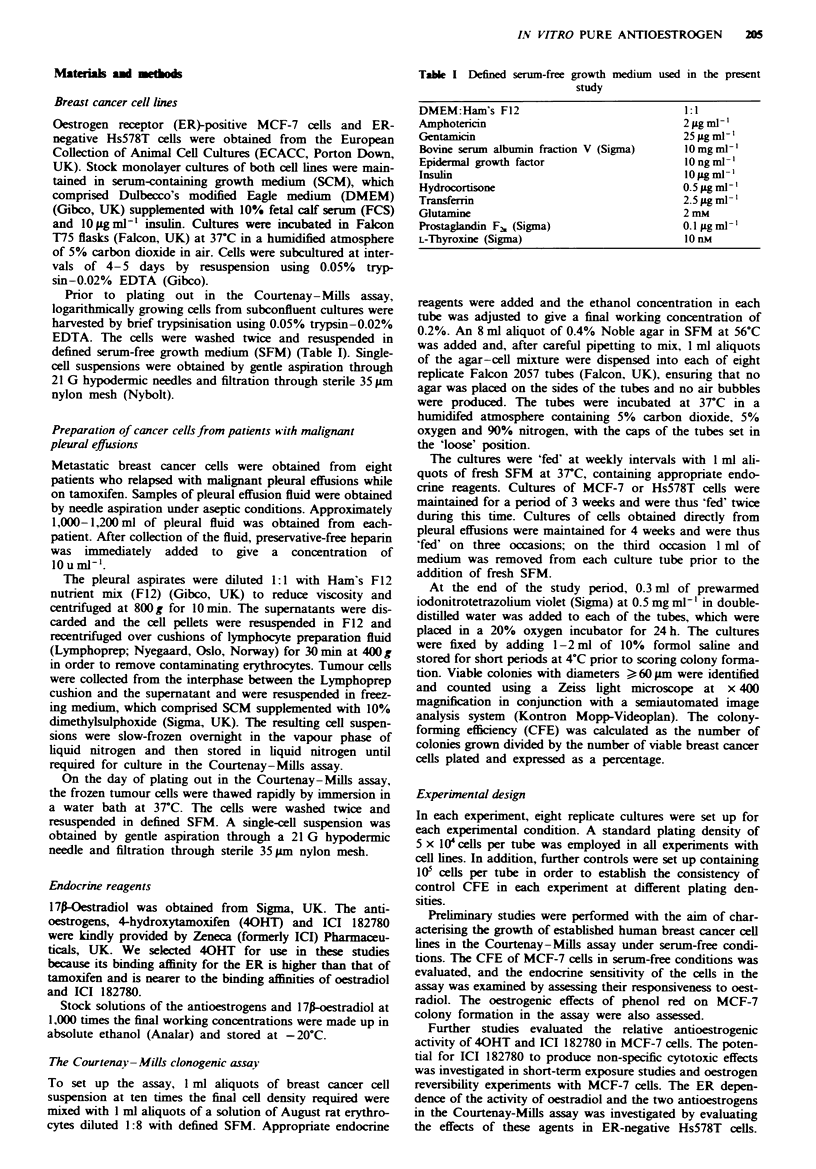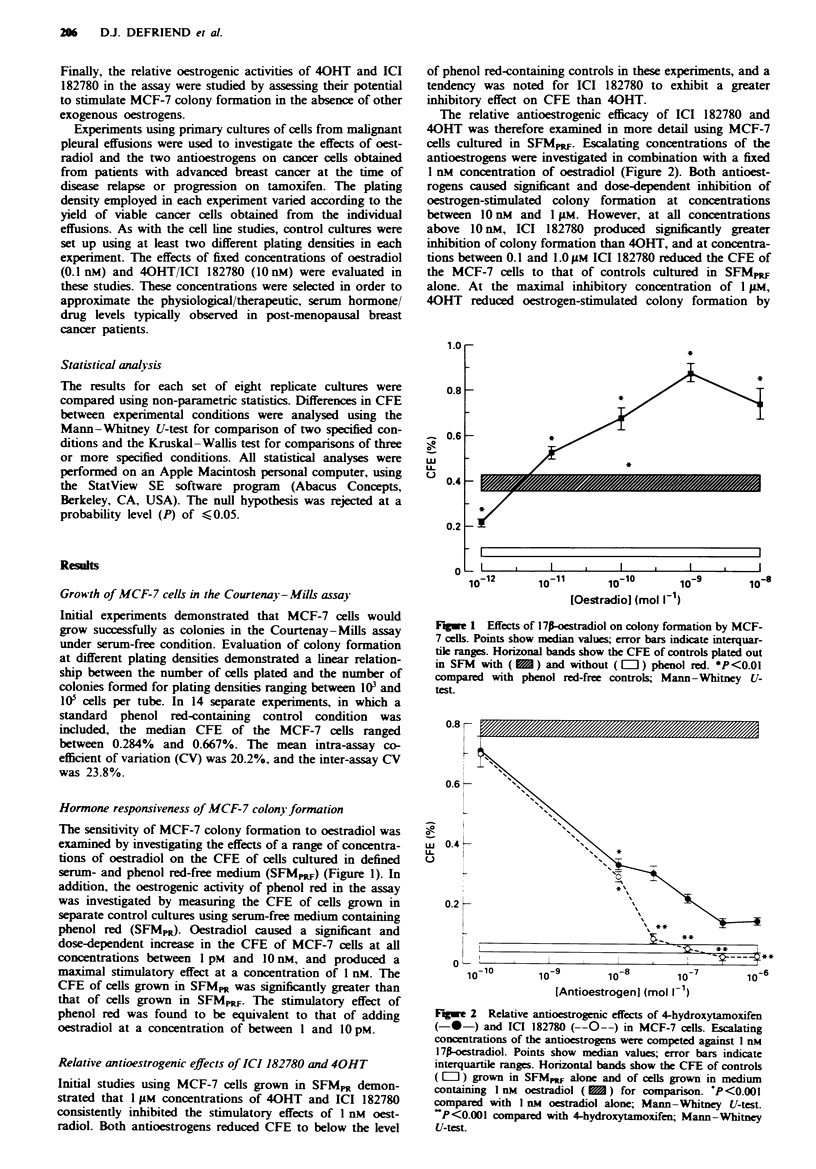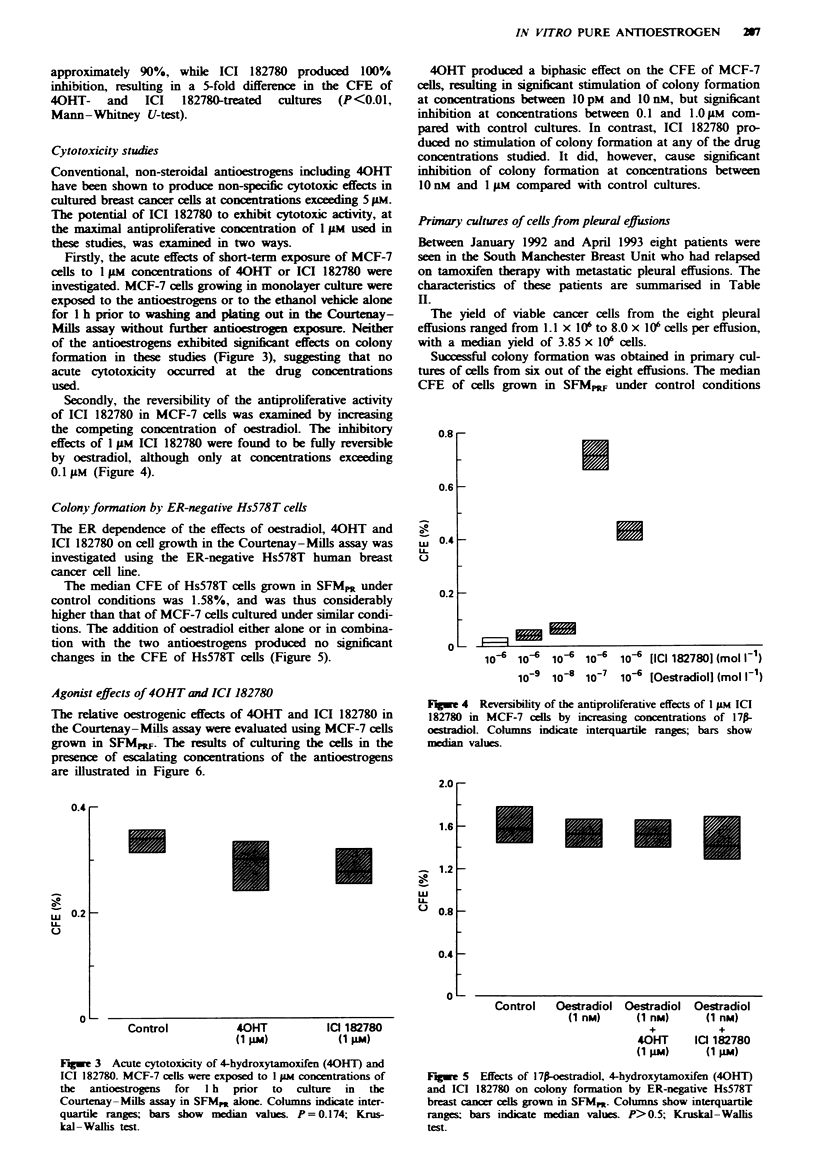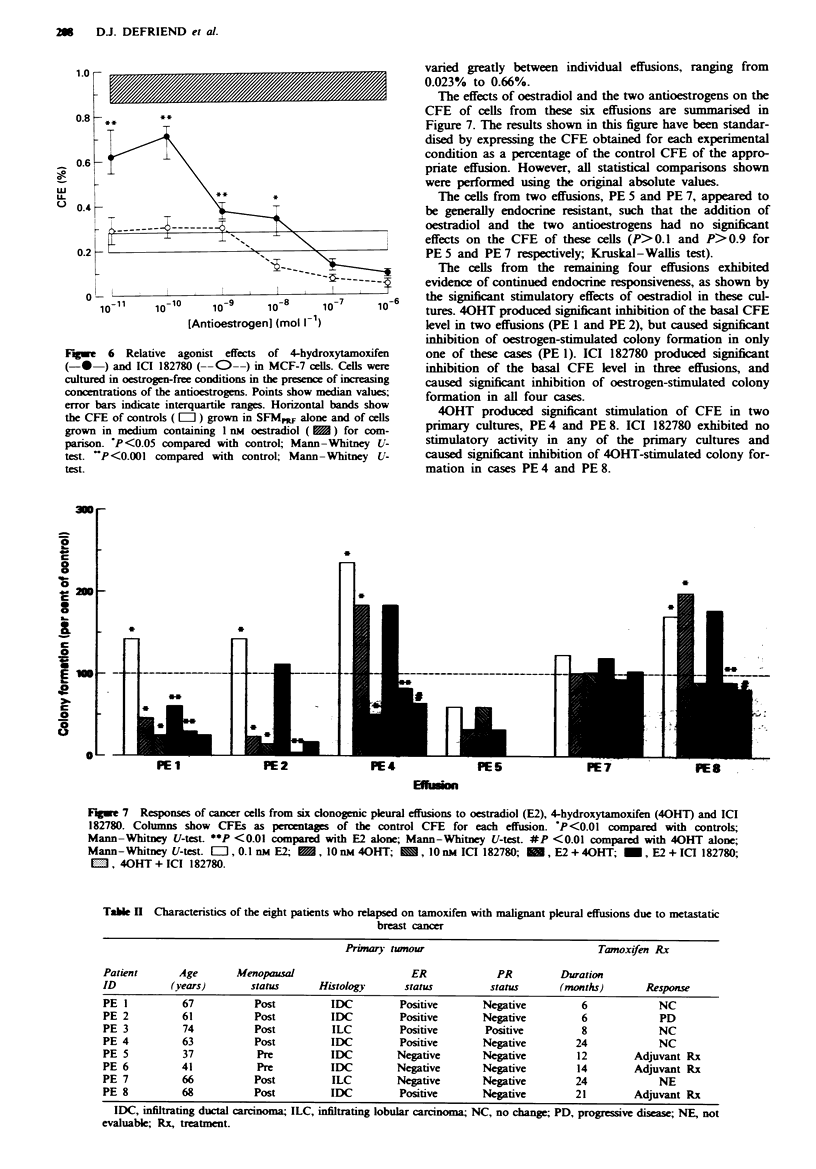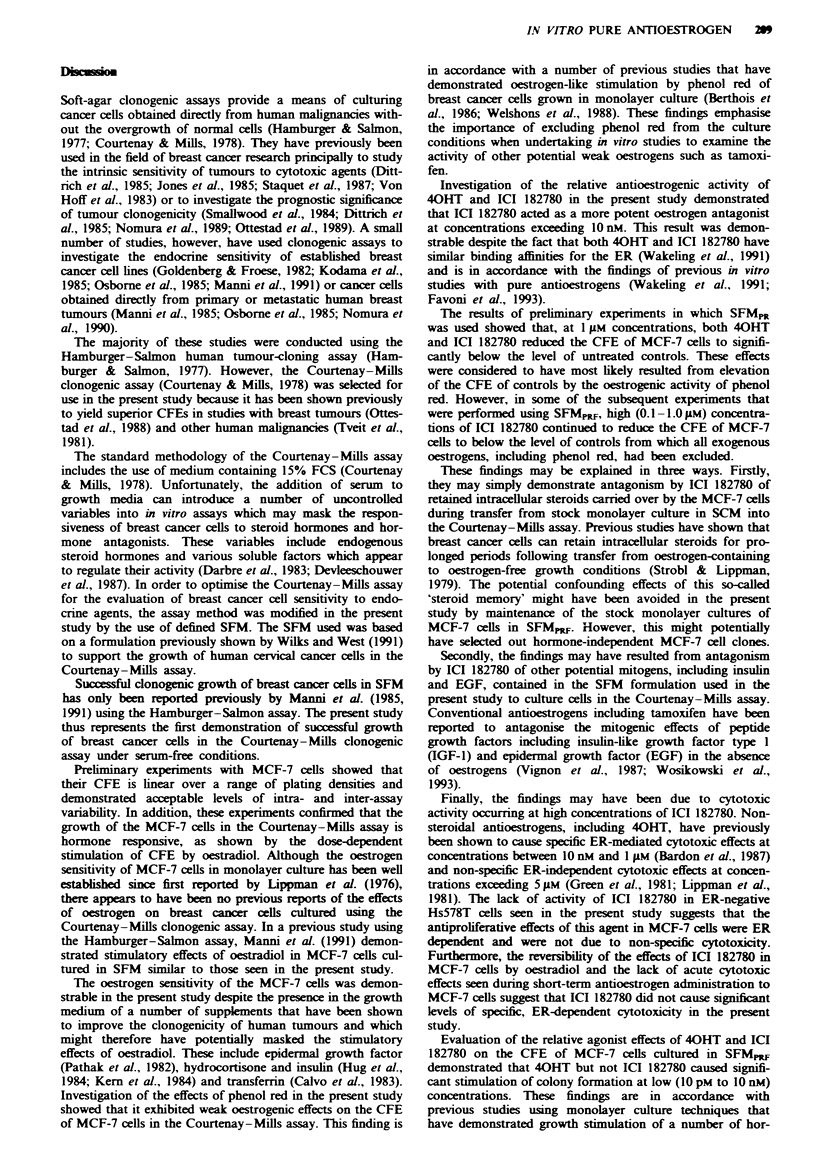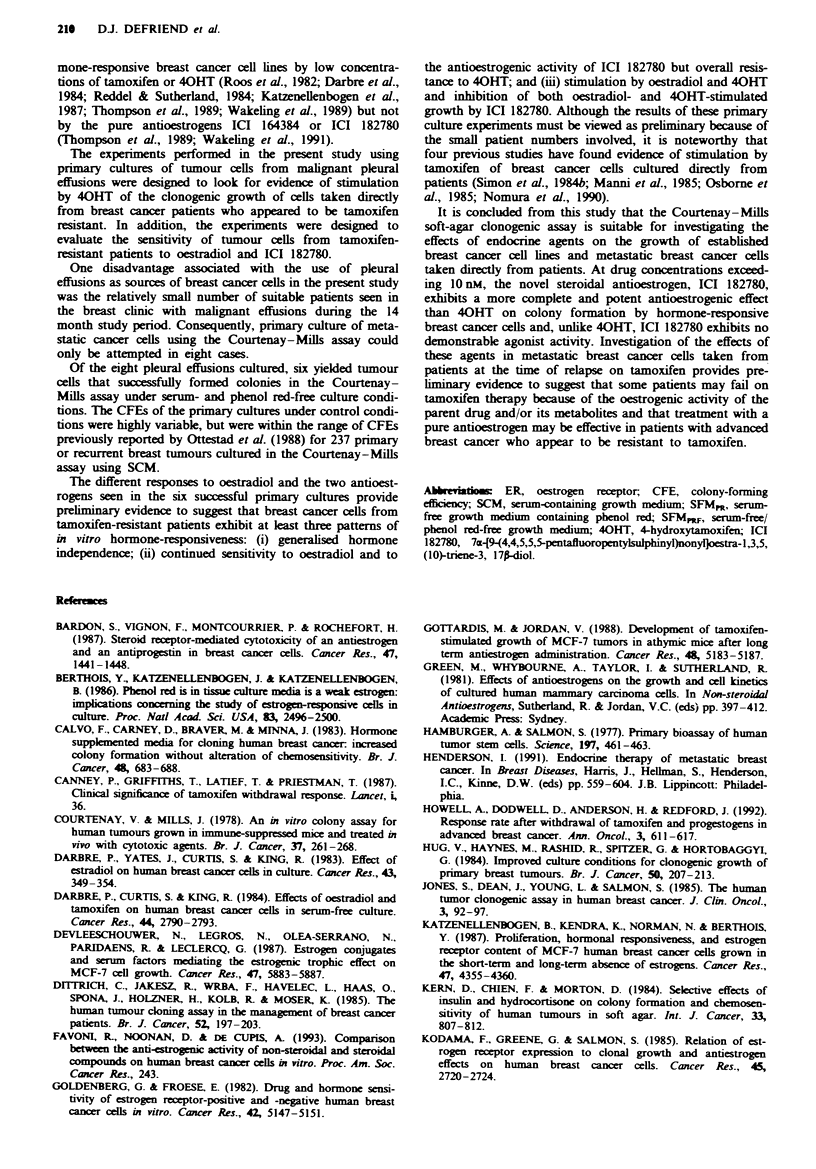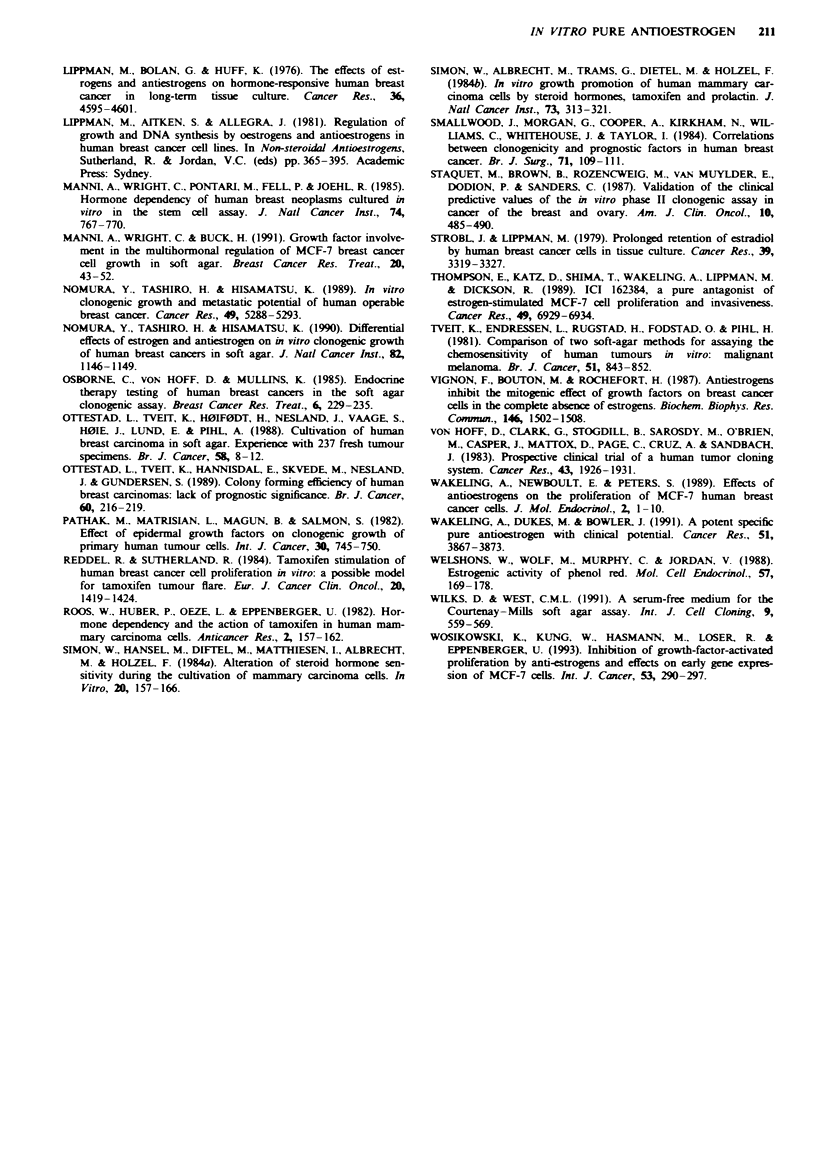# Effects of 4-hydroxytamoxifen and a novel pure antioestrogen (ICI 182780) on the clonogenic growth of human breast cancer cells in vitro.

**DOI:** 10.1038/bjc.1994.281

**Published:** 1994-08

**Authors:** D. J. DeFriend, E. Anderson, J. Bell, D. P. Wilks, C. M. West, R. E. Mansel, A. Howell

**Affiliations:** University Department of Surgery, University Hospital of South Manchester, UK.

## Abstract

**Images:**


Br. J. Cancer (1994), 70, 204-211 © Macmillan Press Ltd., 1994

### Effects of 4-hydroxytamoxifen and a novel pure antioestrogen (ICI 182780) on the clonogenic growth of human breast cancer cells *in vitro*

D.J. DeFriend[1], E. Anderson[2], J. Bell[2], D.P. Wilks[3], C.M.L. West[3], R.E. Mansel[1] & A. Howell[4]

[1]*University Department of Surgery, University Hospital of South Manchester; Departments of* [2]*Clinical Research,* [3]*Radiobiology and* [4]*Medical Oncology, Christie Hospital and Paterson Institute for Cancer Research, Manchester, UK.*

**Summary** We have investigated the effects on breast cancer cell growth of 4-hydroxytamoxifen (4OHT), a conventional antioestrogen with agonist activity, and 7α-[9-(4,4,5,5,5-pentafluoropentylsulphinyl)nonyl]oestra-1,3,5,(10)-triene-3,17β-diol (ICI 182780), a novel, pure antioestrogen, using established human breast cancer cell lines and cancer cells obtained directly from breast cancer patients with malignant pleural effusions who had relapsed on tamoxifen. The effects of the two agents were assessed using the Courtenay-Mills clonogenic assay, which measures the growth of single cancer cells as colonies suspended in soft agar. The standard assay was modified by the use of defined serum- and phenol red-free growth medium. The growth of oestrogen receptor (ER)-positive MCF-7 cells in the assay was oestrogen responsive. Both antioestrogens inhibited the stimulatory effects of 1 nM oestradiol, but ICI 182780 caused significantly greater inhibition than 4OHT at 0.1-1.0 μM concentrations. In the absence of oestradiol, 4OHT but not ICI 182780 caused significant stimulation of colony formation at low (0.01-1.00 nM) concentrations. Neither antioestrogen had any effects on colony formation by the ER-negative Hs578T cell line. Successful colony formation was obtained in primary cultures from six out of eight malignant effusions. Colony formation was significantly stimulated by 0.1 nM oestradiol in four cases and by 10 nM 4OHT in two cases. In contrast, ICI 182780 exhibited no intrinsic stimulatory activity and significantly inhibited both oestradiol- and 4OHT-stimulated cell growth. We conclude that the agonist activity of 4OHT and other conventional antioestrogens may cause treatment failure in some patients by stimulating breast cancer cell growth. The new, pure antioestrogen ICI 182780 is a more potent oestrogen antagonist than 4OHT and exhibits no growth-stimulatory activity. This agent may therefore offer therapeutic advantages over conventional antioestrogens in patients with advanced breast cancer and may be effective after conventional agents have failed.

Tamoxifen is currently the first-line endocrine treatment of choice for hormone-responsive breast cancer. Although at least one-third of patients with advanced breast cancer initially respond to tamoxifen, almost all eventually relapse while on continuing treatment. The mechanisms underlying these treatment failures remain incompletely understood. While tumour progression to an endocrine-independent phenotype may explain some treatment failures, it is clear from the reported response rates to second-line endocrine therapy (Henderson, 1991) that a significant proportion of breast tumours remain hormone responsive after tamoxifen has failed.

Clinical evidence from studies demonstrating tumour responses to withdrawal of tamoxifen at the time of treatment failure (Canney *et al.*, 1987; Howell *et al.*, 1992) suggests that some patients relapse on tamoxifen because their tumours become stimulated by the agonist (oestrogenic) activity exhibited by tamoxifen and other non-steroidal antioestrogens.

These clinical findings are supported by the results of laboratory studies with established human breast cancer cell lines, showing stimulation of cell growth by tamoxifen both *in vitro* (Darbre *et al.* 1984; Reddel & Sutherland, 1984; Katzenellenbogen *et al.*, 1987; Wakeling *et al.*, 1989) and *in vivo* (Gottardis & Jordan, 1988). However, the relevance of these experimental studies to human breast cancer may be diminished by alterations in endocrine responsiveness that immortalised breast cancer cell lines may undergo during long-term cultivation (Simon *et al.*, 1984*a*). As a consequence, established cancer cell lines may become progressively less representative of the cell population of the tumour from which they originated.

We considered that a more representative experimental model with which to examine the contribution of tamoxifen's oestrogenic activity to treatment failure might be achieved by establishing primary cultures of tumour cells taken directly from breast cancer patients at the time of relapse on tamoxifen. This would permit the effects of tamoxifen to be evaluated directly in breast cancer cells from tumours which appeared clinically to be tamoxifen resistant and compared with the effects of ICI 182780, a new steroidal antioestrogen, which appears to be a pure oestrogen antagonist with no demonstrable agonist activity (Wakeling *et al.*, 1991).

Unfortunately, obtaining tumour cells directly from patients with advanced breast cancer is frequently impractical because many patients relapse at sites which are not readily amenable to surgical biopsy. A previous study by Simon *et al.* (1984*b*) demonstrated that primary monolayer cultures could be established successfully by using tumour cells obtained from malignant pleural effusions in patients with advanced breast cancer who had relapsed in lung or pleura. Using this technique, Simon *et al.* (1984*b*) were able to demonstrate stimulation of tumour cell proliferation by tamoxifen in cultures from four out of ten patients.

Because it may prove difficult to prevent the overgrowth of normal fibroblasts and mesothelial cells using monolayer tissue culture methods, we investigated the relative effects of the two antioestrogens using the Courtenay-Mills clonogenic assay (Courtenay & Mills, 1978), which permits the growth of human cancer cells as colonies suspended in semi-solid culture medium without excessive growth of normal cell contaminants. For the purposes of the present study, the standard protocol of the Courtenay-Mills assay was modified by the use of serum-free growth medium, in order to optimise the culture conditions for demonstrating breast cancer cell responsiveness to oestrogens and antioestrogens.

The aims of the present study were firstly to characterise the growth of human breast cancer cells in the Courtenay-Mills assay under serum-free conditions, using established cell lines, and secondly to utilise the assay to investigate the effects of tamoxifen and ICI 182780 in primary cultures of breast cancer cells taken from patients with advanced breast cancer who had relapsed on tamoxifen.

Correspondence: D.J. DeFriend, Department of Surgery, TU5, University Hospital of South Manchester, Nell Lane, West Didsbury, Manchester M20 8LR, UK.
Received 14 October 1993; and in revised form 23 February 1994.

### Materials and methods

### *Breast cancer cell lines*

Oestrogen receptor (ER)-positive MCF-7 cells and ER-negative Hs578T cells were obtained from the European Collection of Animal Cell Cultures (ECACC, Porton Down, UK). Stock monolayer cultures of both cell lines were maintained in serum-containing growth medium (SCM), which comprised Dulbecco's modified Eagle medium (DMEM) (Gibco, UK) supplemented with 10% fetal calf serum (FCS) and 10 $\mu g\ ml^{-1}$ insulin. Cultures were incubated in Falcon T75 flasks (Falcon, UK) at 37°C in a humidified atmosphere of 5% carbon dioxide in air. Cells were subcultured at intervals of 4-5 days by resuspension using 0.05% trypsin-0.02% EDTA (Gibco).

Prior to plating out in the Courtenay-Mills assay, logarithmically growing cells from subconfluent cultures were harvested by brief trypsinisation using 0.05% trypsin-0.02% EDTA. The cells were washed twice and resuspended in defined serum-free growth medium (SFM) (Table I). Single-cell suspensions were obtained by gentle aspiration through 21 G hypodermic needles and filtration through sterile 35 $\mu m$ nylon mesh (Nybolt).

### *Preparation of cancer cells from patients with malignant pleural effusions*

Metastatic breast cancer cells were obtained from eight patients who relapsed with malignant pleural effusions while on tamoxifen. Samples of pleural effusion fluid were obtained by needle aspiration under aseptic conditions. Approximately 1,000-1,200 ml of pleural fluid was obtained from each-patient. After collection of the fluid, preservative-free heparin was immediately added to give a concentration of 10 u $ml^{-1}$.

The pleural aspirates were diluted 1:1 with Ham's F12 nutrient mix (F12) (Gibco, UK) to reduce viscosity and centrifuged at 800 *g* for 10 min. The supernatants were discarded and the cell pellets were resuspended in F12 and recentrifuged over cushions of lymphocyte preparation fluid (Lymphoprep; Nyegaard, Oslo, Norway) for 30 min at 400 *g* in order to remove contaminating erythrocytes. Tumour cells were collected from the interphase between the Lymphoprep cushion and the supernatant and were resuspended in freezing medium, which comprised SCM supplemented with 10% dimethylsulphoxide (Sigma, UK). The resulting cell suspensions were slow-frozen overnight in the vapour phase of liquid nitrogen and then stored in liquid nitrogen until required for culture in the Courtenay-Mills assay.

On the day of plating out in the Courtenay-Mills assay, the frozen tumour cells were thawed rapidly by immersion in a water bath at 37°C. The cells were washed twice and resuspended in defined SFM. A single-cell suspension was obtained by gentle aspiration through a 21 G hypodermic needle and filtration through sterile 35 $\mu m$ nylon mesh.

### *Endocrine reagents*

17β-Oestradiol was obtained from Sigma, UK. The antioestrogens, 4-hydroxytamoxifen (4OHT) and ICI 182780 were kindly provided by Zeneca (formerly ICI) Pharmaceuticals, UK. We selected 4OHT for use in these studies because its binding affinity for the ER is higher than that of tamoxifen and is nearer to the binding affinities of oestradiol and ICI 182780.

Stock solutions of the antioestrogens and 17β-oestradiol at 1,000 times the final working concentrations were made up in absolute ethanol (Analar) and stored at −20°C.

### *The Courtenay-Mills clonogenic assay*

To set up the assay, 1 ml aliquots of breast cancer cell suspension at ten times the final cell density required were mixed with 1 ml aliquots of a solution of August rat erythrocytes diluted 1:8 with defined SFM. Appropriate endocrine reagents were added and the ethanol concentration in each tube was adjusted to give a final working concentration of 0.2%. An 8 ml aliquot of 0.4% Noble agar in SFM at 56°C was added and, after careful pipetting to mix, 1 ml aliquots of the agar-cell mixture were dispensed into each of eight replicate Falcon 2057 tubes (Falcon, UK), ensuring that no agar was placed on the sides of the tubes and no air bubbles were produced. The tubes were incubated at 37°C in a humidifed atmosphere containing 5% carbon dioxide, 5% oxygen and 90% nitrogen, with the caps of the tubes set in the 'loose' position.

The cultures were 'fed' at weekly intervals with 1 ml aliquots of fresh SFM at 37°C, containing appropriate endocrine reagents. Cultures of MCF-7 or Hs578T cells were maintained for a period of 3 weeks and were thus 'fed' twice during this time. Cultures of cells obtained directly from pleural effusions were maintained for 4 weeks and were thus 'fed' on three occasions; on the third occasion 1 ml of medium was removed from each culture tube prior to the addition of fresh SFM.

At the end of the study period, 0.3 ml of prewarmed iodonitrotetrazolium violet (Sigma) at 0.5 mg $ml^{-1}$ in double-distilled water was added to each of the tubes, which were placed in a 20% oxygen incubator for 24 h. The cultures were fixed by adding 1-2 ml of 10% formol saline and stored for short periods at 4°C prior to scoring colony formation. Viable colonies with diameters ⩾60 $\mu m$ were identified and counted using a Zeiss light microscope at ×400 magnification in conjunction with a semiautomated image analysis system (Kontron Mopp-Videoplan). The colony-forming efficiency (CFE) was calculated as the number of colonies grown divided by the number of viable breast cancer cells plated and expressed as a percentage.

**Table I** Defined serum-free growth medium used in the present study

| | |
|---|---|
| DMEM:Ham's F12 | 1:1 |
| Amphotericin | 2 $\mu g\ ml^{-1}$ |
| Gentamicin | 25 $\mu g\ ml^{-1}$ |
| Bovine serum albumin fraction V (Sigma) | 10 mg $ml^{-1}$ |
| Epidermal growth factor | 10 ng $ml^{-1}$ |
| Insulin | 10 $\mu g\ ml^{-1}$ |
| Hydrocortisone | 0.5 $\mu g\ ml^{-1}$ |
| Transferrin | 2.5 $\mu g\ ml^{-1}$ |
| Glutamine | 2 mM |
| Prostaglandin $F_{2\alpha}$ (Sigma) | 0.1 $\mu g\ ml^{-1}$ |
| L-Thyroxine (Sigma) | 10 nM |

### *Experimental design*

In each experiment, eight replicate cultures were set up for each experimental condition. A standard plating density of $5 \times 10^4$ cells per tube was employed in all experiments with cell lines. In addition, further controls were set up containing $10^5$ cells per tube in order to establish the consistency of control CFE in each experiment at different plating densities.

Preliminary studies were performed with the aim of characterising the growth of established human breast cancer cell lines in the Courtenay-Mills assay under serum-free conditions. The CFE of MCF-7 cells in serum-free conditions was evaluated, and the endocrine sensitivity of the cells in the assay was examined by assessing their responsiveness to oestradiol. The oestrogenic effects of phenol red on MCF-7 colony formation in the assay were also assessed.

Further studies evaluated the relative antioestrogenic activity of 4OHT and ICI 182780 in MCF-7 cells. The potential for ICI 182780 to produce non-specific cytotoxic effects was investigated in short-term exposure studies and oestrogen reversibility experiments with MCF-7 cells. The ER dependence of the activity of oestradiol and the two antioestrogens in the Courtenay-Mills assay was investigated by evaluating the effects of these agents in ER-negative Hs578T cells.

Finally, the relative oestrogenic activities of 4OHT and ICI 182780 in the assay were studied by assessing their potential to stimulate MCF-7 colony formation in the absence of other exogenous oestrogens.

Experiments using primary cultures of cells from malignant pleural effusions were used to investigate the effects of oestradiol and the two antioestrogens on cancer cells obtained from patients with advanced breast cancer at the time of disease relapse or progression on tamoxifen. The plating density employed in each experiment varied according to the yield of viable cancer cells obtained from the individual effusions. As with the cell line studies, control cultures were set up using at least two different plating densities in each experiment. The effects of fixed concentrations of oestradiol (0.1 nM) and 4OHT/ICI 182780 (10 nM) were evaluated in these studies. These concentrations were selected in order to approximate the physiological/therapeutic, serum hormone/drug levels typically observed in post-menopausal breast cancer patients.

### *Statistical analysis*

The results for each set of eight replicate cultures were compared using non-parametric statistics. Differences in CFE between experimental conditions were analysed using the Mann-Whitney *U*-test for comparison of two specified conditions and the Kruskal-Wallis test for comparisons of three or more specified conditions. All statistical analyses were performed on an Apple Macintosh personal computer, using the StatView SE software program (Abacus Concepts, Berkeley, CA, USA). The null hypothesis was rejected at a probability level ($P$) of $\leqslant 0.05$.

### Results

### *Growth of MCF-7 cells in the Courtenay-Mills assay*

Initial experiments demonstrated that MCF-7 cells would grow successfully as colonies in the Courtenay-Mills assay under serum-free condition. Evaluation of colony formation at different plating densities demonstrated a linear relationship between the number of cells plated and the number of colonies formed for plating densities ranging between $10^3$ and $10^5$ cells per tube. In 14 separate experiments, in which a standard phenol red-containing control condition was included, the median CFE of the MCF-7 cells ranged between 0.284% and 0.667%. The mean intra-assay coefficient of variation (CV) was 20.2%, and the inter-assay CV was 23.8%.

### *Hormone responsiveness of MCF-7 colony formation*

The sensitivity of MCF-7 colony formation to oestradiol was examined by investigating the effects of a range of concentrations of oestradiol on the CFE of cells cultured in defined serum- and phenol red-free medium ($SFM_{PRF}$) (Figure 1). In addition, the oestrogenic activity of phenol red in the assay was investigated by measuring the CFE of cells grown in separate control cultures using serum-free medium containing phenol red ($SFM_{PR}$). Oestradiol caused a significant and dose-dependent increase in the CFE of MCF-7 cells at all concentrations between 1 pM and 10 nM, and produced a maximal stimulatory effect at a concentration of 1 nM. The CFE of cells grown in $SFM_{PR}$ was significantly greater than that of cells grown in $SFM_{PRF}$. The stimulatory effect of phenol red was found to be equivalent to that of adding oestradiol at a concentration of between 1 and 10 pM.

### *Relative antioestrogenic effects of ICI 182780 and 4OHT*

Initial studies using MCF-7 cells grown in $SFM_{PR}$ demonstrated that 1 µM concentrations of 4OHT and ICI 182780 consistently inhibited the stimulatory effects of 1 nM oestradiol. Both antioestrogens reduced CFE to below the level of phenol red-containing controls in these experiments, and a tendency was noted for ICI 182780 to exhibit a greater inhibitory effect on CFE than 4OHT.

The relative antioestrogenic efficacy of ICI 182780 and 4OHT was therefore examined in more detail using MCF-7 cells cultured in $SFM_{PRF}$. Escalating concentrations of the antioestrogens were investigated in combination with a fixed 1 nM concentration of oestradiol (Figure 2). Both antioestrogens caused significant and dose-dependent inhibition of oestrogen-stimulated colony formation at concentrations between 10 nM and 1 µM. However, at all concentrations above 10 nM, ICI 182780 produced significantly greater inhibition of colony formation than 4OHT, and at concentrations between 0.1 and 1.0 µM ICI 182780 reduced the CFE of the MCF-7 cells to that of controls cultured in $SFM_{PRF}$ alone. At the maximal inhibitory concentration of 1 µM, 4OHT reduced oestrogen-stimulated colony formation by

**Figure 1** Effects of 17β-oestradiol on colony formation by MCF-7 cells. Points show median values; error bars indicate interquartile ranges. Horizonal bands show the CFE of controls plated out in SFM with ( ▨ ) and without ( ☐ ) phenol red. *$P<0.01$ compared with phenol red-free controls; Mann-Whitney *U*-test.

**Figure 2** Relative antioestrogenic effects of 4-hydroxytamoxifen (—●—) and ICI 182780 (--○--) in MCF-7 cells. Escalating concentrations of the antioestrogens were competed against 1 nM 17β-oestradiol. Points show median values; error bars indicate interquartile ranges. Horizontal bands show the CFE of controls ( ☐ ) grown in $SFM_{PRF}$ alone and of cells grown in medium containing 1 nM oestradiol ( ▨ ) for comparison. *$P<0.001$ compared with 1 nM oestradiol alone; Mann-Whitney *U*-test. **$P<0.001$ compared with 4-hydroxytamoxifen; Mann-Whitney *U*-test.

approximately 90%, while ICI 182780 produced 100% inhibition, resulting in a 5-fold difference in the CFE of 4OHT- and ICI 182780-treated cultures ($P<0.01$, Mann–Whitney *U*-test).

*Cytotoxicity studies*

Conventional, non-steroidal antioestrogens including 4OHT have been shown to produce non-specific cytotoxic effects in cultured breast cancer cells at concentrations exceeding 5 μM. The potential of ICI 182780 to exhibit cytotoxic activity, at the maximal antiproliferative concentration of 1 μM used in these studies, was examined in two ways.

Firstly, the acute effects of short-term exposure of MCF-7 cells to 1 μM concentrations of 4OHT or ICI 182780 were investigated. MCF-7 cells growing in monolayer culture were exposed to the antioestrogens or to the ethanol vehicle alone for 1 h prior to washing and plating out in the Courtenay–Mills assay without further antioestrogen exposure. Neither of the antioestrogens exhibited significant effects on colony formation in these studies (Figure 3), suggesting that no acute cytotoxicity occurred at the drug concentrations used.

Secondly, the reversibility of the antiproliferative activity of ICI 182780 in MCF-7 cells was examined by increasing the competing concentration of oestradiol. The inhibitory effects of 1 μM ICI 182780 were found to be fully reversible by oestradiol, although only at concentrations exceeding 0.1 μM (Figure 4).

*Colony formation by ER-negative Hs578T cells*

The ER dependence of the effects of oestradiol, 4OHT and ICI 182780 on cell growth in the Courtenay–Mills assay was investigated using the ER-negative Hs578T human breast cancer cell line.

The median CFE of Hs578T cells grown in $SFM_{PR}$ under control conditions was 1.58%, and was thus considerably higher than that of MCF-7 cells cultured under similar conditions. The addition of oestradiol either alone or in combination with the two antioestrogens produced no significant changes in the CFE of Hs578T cells (Figure 5).

*Agonist effects of 4OHT and ICI 182780*

The relative oestrogenic effects of 4OHT and ICI 182780 in the Courtenay–Mills assay were evaluated using MCF-7 cells grown in $SFM_{PRF}$. The results of culturing the cells in the presence of escalating concentrations of the antioestrogens are illustrated in Figure 6.

**Figure 3** Acute cytotoxicity of 4-hydroxytamoxifen (4OHT) and ICI 182780. MCF-7 cells were exposed to 1 μM concentrations of the antioestrogens for 1 h prior to culture in the Courtenay–Mills assay in $SFM_{PR}$ alone. Columns indicate interquartile ranges; bars show median values. $P=0.174$; Kruskal–Wallis test.

4OHT produced a biphasic effect on the CFE of MCF-7 cells, resulting in significant stimulation of colony formation at concentrations between 10 pM and 10 nM, but significant inhibition at concentrations between 0.1 and 1.0 μM compared with control cultures. In contrast, ICI 182780 produced no stimulation of colony formation at any of the drug concentrations studied. It did, however, cause significant inhibition of colony formation at concentrations between 10 nM and 1 μM compared with control cultures.

*Primary cultures of cells from pleural effusions*

Between January 1992 and April 1993 eight patients were seen in the South Manchester Breast Unit who had relapsed on tamoxifen therapy with metastatic pleural effusions. The characteristics of these patients are summarised in Table II.

The yield of viable cancer cells from the eight pleural effusions ranged from $1.1 \times 10^6$ to $8.0 \times 10^6$ cells per effusion, with a median yield of $3.85 \times 10^6$ cells.

Successful colony formation was obtained in primary cultures of cells from six out of the eight effusions. The median CFE of cells grown in $SFM_{PRF}$ under control conditions

**Figure 4** Reversibility of the antiproliferative effects of 1 μM ICI 182780 in MCF-7 cells by increasing concentrations of 17β-oestradiol. Columns indicate interquartile ranges; bars show median values.

**Figure 5** Effects of 17β-oestradiol, 4-hydroxytamoxifen (4OHT) and ICI 182780 on colony formation by ER-negative Hs578T breast cancer cells grown in $SFM_{PR}$. Columns show interquartile ranges; bars indicate median values. $P>0.5$; Kruskal–Wallis test.

**Figure 6** Relative agonist effects of 4-hydroxytamoxifen (—●—) and ICI 182780 (--○--) in MCF-7 cells. Cells were cultured in oestrogen-free conditions in the presence of increasing concentrations of the antioestrogens. Points show median values; error bars indicate interquartile ranges. Horizontal bands show the CFE of controls (□) grown in $SFM_{PRF}$ alone and of cells grown in medium containing 1 nM oestradiol (▨) for comparison. *$P<0.05$ compared with control; Mann–Whitney *U*-test. **$P<0.001$ compared with control; Mann–Whitney *U*-test.

varied greatly between individual effusions, ranging from 0.023% to 0.66%.

The effects of oestradiol and the two antioestrogens on the CFE of cells from these six effusions are summarised in Figure 7. The results shown in this figure have been standardised by expressing the CFE obtained for each experimental condition as a percentage of the control CFE of the appropriate effusion. However, all statistical comparisons shown were performed using the original absolute values.

The cells from two effusions, PE 5 and PE 7, appeared to be generally endocrine resistant, such that the addition of oestradiol and the two antioestrogens had no significant effects on the CFE of these cells ($P>0.1$ and $P>0.9$ for PE 5 and PE 7 respectively; Kruskal–Wallis test).

The cells from the remaining four effusions exhibited evidence of continued endocrine responsiveness, as shown by the significant stimulatory effects of oestradiol in these cultures. 4OHT produced significant inhibition of the basal CFE level in two effusions (PE 1 and PE 2), but caused significant inhibition of oestrogen-stimulated colony formation in only one of these cases (PE 1). ICI 182780 produced significant inhibition of the basal CFE level in three effusions, and caused significant inhibition of oestrogen-stimulated colony formation in all four cases.

4OHT produced significant stimulation of CFE in two primary cultures, PE 4 and PE 8. ICI 182780 exhibited no stimulatory activity in any of the primary cultures and caused significant inhibition of 4OHT-stimulated colony formation in cases PE 4 and PE 8.

**Figure 7** Responses of cancer cells from six clonogenic pleural effusions to oestradiol (E2), 4-hydroxytamoxifen (4OHT) and ICI 182780. Columns show CFEs as percentages of the control CFE for each effusion. *$P<0.01$ compared with controls; Mann–Whitney *U*-test. **$P<0.01$ compared with E2 alone; Mann–Whitney *U*-test. #$P<0.01$ compared with 4OHT alone; Mann–Whitney *U*-test. □, 0.1 nM E2; ▨, 10 nM 4OHT; ▧, 10 nM ICI 182780; ▩, E2 + 4OHT; ■, E2 + ICI 182780; ▤, 4OHT + ICI 182780.

**Table II** Characteristics of the eight patients who relapsed on tamoxifen with malignant pleural effusions due to metastatic breast cancer

| | | | *Primary tumour* | | | *Tamoxifen Rx* | |
|---|---|---|---|---|---|---|---|
| *Patient ID* | *Age (years)* | *Menopausal status* | *Histology* | *ER status* | *PR status* | *Duration (months)* | *Response* |
| PE 1 | 67 | Post | IDC | Positive | Negative | 6 | NC |
| PE 2 | 61 | Post | IDC | Positive | Negative | 6 | PD |
| PE 3 | 74 | Post | ILC | Positive | Positive | 8 | NC |
| PE 4 | 63 | Post | IDC | Positive | Negative | 24 | NC |
| PE 5 | 37 | Pre | IDC | Negative | Negative | 12 | Adjuvant Rx |
| PE 6 | 41 | Pre | IDC | Negative | Negative | 14 | Adjuvant Rx |
| PE 7 | 66 | Post | ILC | Negative | Negative | 24 | NE |
| PE 8 | 68 | Post | IDC | Positive | Negative | 21 | Adjuvant Rx |

IDC, infiltrating ductal carcinoma; ILC, infiltrating lobular carcinoma; NC, no change; PD, progressive disease; NE, not evaluable; Rx, treatment.

### Discussion

Soft-agar clonogenic assays provide a means of culturing cancer cells obtained directly from human malignancies without the overgrowth of normal cells (Hamburger & Salmon, 1977; Courtenay & Mills, 1978). They have previously been used in the field of breast cancer research principally to study the intrinsic sensitivity of tumours to cytotoxic agents (Dittrich *et al.*, 1985; Jones *et al.*, 1985; Staquet *et al.*, 1987; Von Hoff *et al.*, 1983) or to investigate the prognostic significance of tumour clonogenicity (Smallwood *et al.*, 1984; Dittrich *et al.*, 1985; Nomura *et al.*, 1989; Ottestad *et al.*, 1989). A small number of studies, however, have used clonogenic assays to investigate the endocrine sensitivity of established breast cancer cell lines (Goldenberg & Froese, 1982; Kodama *et al.*, 1985; Osborne *et al.*, 1985; Manni *et al.*, 1991) or cancer cells obtained directly from primary or metastatic human breast tumours (Manni *et al.*, 1985; Osborne *et al.*, 1985; Nomura *et al.*, 1990).

The majority of these studies were conducted using the Hamburger-Salmon human tumour-cloning assay (Hamburger & Salmon, 1977). However, the Courtenay-Mills clonogenic assay (Courtenay & Mills, 1978) was selected for use in the present study because it has been shown previously to yield superior CFEs in studies with breast tumours (Ottestad *et al.*, 1988) and other human malignancies (Tveit *et al.*, 1981).

The standard methodology of the Courtenay-Mills assay includes the use of medium containing 15% FCS (Courtenay & Mills, 1978). Unfortunately, the addition of serum to growth media can introduce a number of uncontrolled variables into *in vitro* assays which may mask the responsiveness of breast cancer cells to steroid hormones and hormone antagonists. These variables include endogenous steroid hormones and various soluble factors which appear to regulate their activity (Darbre *et al.*, 1983; Devleeschouwer *et al.*, 1987). In order to optimise the Courtenay-Mills assay for the evaluation of breast cancer cell sensitivity to endocrine agents, the assay method was modified in the present study by the use of defined SFM. The SFM used was based on a formulation previously shown by Wilks and West (1991) to support the growth of human cervical cancer cells in the Courtenay-Mills assay.

Successful clonogenic growth of breast cancer cells in SFM has only been reported previously by Manni *et al.* (1985, 1991) using the Hamburger-Salmon assay. The present study thus represents the first demonstration of successful growth of breast cancer cells in the Courtenay-Mills clonogenic assay under serum-free conditions.

Preliminary experiments with MCF-7 cells showed that their CFE is linear over a range of plating densities and demonstrated acceptable levels of intra- and inter-assay variability. In addition, these experiments confirmed that the growth of the MCF-7 cells in the Courtenay-Mills assay is hormone responsive, as shown by the dose-dependent stimulation of CFE by oestradiol. Although the oestrogen sensitivity of MCF-7 cells in monolayer culture has been well established since first reported by Lippman *et al.* (1976), there appears to have been no previous reports of the effects of oestrogen on breast cancer cells cultured using the Courtenay-Mills clonogenic assay. In a previous study using the Hamburger-Salmon assay, Manni *et al.* (1991) demonstrated stimulatory effects of oestradiol in MCF-7 cells cultured in SFM similar to those seen in the present study.

The oestrogen sensitivity of the MCF-7 cells was demonstrable in the present study despite the presence in the growth medium of a number of supplements that have been shown to improve the clonogenicity of human tumours and which might therefore have potentially masked the stimulatory effects of oestradiol. These include epidermal growth factor (Pathak *et al.*, 1982), hydrocortisone and insulin (Hug *et al.*, 1984; Kern *et al.*, 1984) and transferrin (Calvo *et al.*, 1983). Investigation of the effects of phenol red in the present study showed that it exhibited weak oestrogenic effects on the CFE of MCF-7 cells in the Courtenay-Mills assay. This finding is in accordance with a number of previous studies that have demonstrated oestrogen-like stimulation by phenol red of breast cancer cells grown in monolayer culture (Berthois *et al.*, 1986; Welshons *et al.*, 1988). These findings emphasise the importance of excluding phenol red from the culture conditions when undertaking *in vitro* studies to examine the activity of other potential weak oestrogens such as tamoxifen.

Investigation of the relative antioestrogenic activity of 4OHT and ICI 182780 in the present study demonstrated that ICI 182780 acted as a more potent oestrogen antagonist at concentrations exceeding 10 nM. This result was demonstrable despite the fact that both 4OHT and ICI 182780 have similar binding affinities for the ER (Wakeling *et al.*, 1991) and is in accordance with the findings of previous *in vitro* studies with pure antioestrogens (Wakeling *et al.*, 1991; Favoni *et al.*, 1993).

The results of preliminary experiments in which $SFM_{PR}$ was used showed that, at 1 μM concentrations, both 4OHT and ICI 182780 reduced the CFE of MCF-7 cells to significantly below the level of untreated controls. These effects were considered to have most likely resulted from elevation of the CFE of controls by the oestrogenic activity of phenol red. However, in some of the subsequent experiments that were performed using $SFM_{PRF}$, high (0.1-1.0 μM) concentrations of ICI 182780 continued to reduce the CFE of MCF-7 cells to below the level of controls from which all exogenous oestrogens, including phenol red, had been excluded.

These findings may be explained in three ways. Firstly, they may simply demonstrate antagonism by ICI 182780 of retained intracellular steroids carried over by the MCF-7 cells during transfer from stock monolayer culture in SCM into the Courtenay-Mills assay. Previous studies have shown that breast cancer cells can retain intracellular steroids for prolonged periods following transfer from oestrogen-containing to oestrogen-free growth conditions (Strobl & Lippman, 1979). The potential confounding effects of this so-called 'steroid memory' might have been avoided in the present study by maintenance of the stock monolayer cultures of MCF-7 cells in $SFM_{PRF}$. However, this might potentially have selected out hormone-independent MCF-7 cell clones.

Secondly, the findings may have resulted from antagonism by ICI 182780 of other potential mitogens, including insulin and EGF, contained in the SFM formulation used in the present study to culture cells in the Courtenay-Mills assay. Conventional antioestrogens including tamoxifen have been reported to antagonise the mitogenic effects of peptide growth factors including insulin-like growth factor type 1 (IGF-1) and epidermal growth factor (EGF) in the absence of oestrogens (Vignon *et al.*, 1987; Wosikowski *et al.*, 1993).

Finally, the findings may have been due to cytotoxic activity occurring at high concentrations of ICI 182780. Non-steroidal antioestrogens, including 4OHT, have previously been shown to cause specific ER-mediated cytotoxic effects at concentrations between 10 nM and 1 μM (Bardon *et al.*, 1987) and non-specific ER-independent cytotoxic effects at concentrations exceeding 5 μM (Green *et al.*, 1981; Lippman *et al.*, 1981). The lack of activity of ICI 182780 in ER-negative Hs578T cells seen in the present study suggests that the antiproliferative effects of this agent in MCF-7 cells were ER dependent and were not due to non-specific cytotoxicity. Furthermore, the reversibility of the effects of ICI 182780 in MCF-7 cells by oestradiol and the lack of acute cytotoxic effects seen during short-term antioestrogen administration to MCF-7 cells suggest that ICI 182780 did not cause significant levels of specific, ER-dependent cytotoxicity in the present study.

Evaluation of the relative agonist effects of 4OHT and ICI 182780 on the CFE of MCF-7 cells cultured in $SFM_{PRF}$ demonstrated that 4OHT but not ICI 182780 caused significant stimulation of colony formation at low (10 pM to 10 nM) concentrations. These findings are in accordance with previous studies using monolayer culture techniques that have demonstrated growth stimulation of a number of hormone-responsive breast cancer cell lines by low concentrations of tamoxifen or 4OHT (Roos *et al.*, 1982; Darbre *et al.*, 1984; Reddel & Sutherland, 1984; Katzenellenbogen *et al.*, 1987; Thompson *et al.*, 1989; Wakeling *et al.*, 1989) but not by the pure antioestrogens ICI 164384 or ICI 182780 (Thompson *et al.*, 1989; Wakeling *et al.*, 1991).

The experiments performed in the present study using primary cultures of tumour cells from malignant pleural effusions were designed to look for evidence of stimulation by 4OHT of the clonogenic growth of cells taken directly from breast cancer patients who appeared to be tamoxifen resistant. In addition, the experiments were designed to evaluate the sensitivity of tumour cells from tamoxifen-resistant patients to oestradiol and ICI 182780.

One disadvantage associated with the use of pleural effusions as sources of breast cancer cells in the present study was the relatively small number of suitable patients seen in the breast clinic with malignant effusions during the 14 month study period. Consequently, primary culture of metastatic cancer cells using the Courtenay-Mills assay could only be attempted in eight cases.

Of the eight pleural effusions cultured, six yielded tumour cells that successfully formed colonies in the Courtenay-Mills assay under serum- and phenol red-free culture conditions. The CFEs of the primary cultures under control conditions were highly variable, but were within the range of CFEs previously reported by Ottestad *et al.* (1988) for 237 primary or recurrent breast tumours cultured in the Courtenay-Mills assay using SCM.

The different responses to oestradiol and the two antioestrogens seen in the six successful primary cultures provide preliminary evidence to suggest that breast cancer cells from tamoxifen-resistant patients exhibit at least three patterns of *in vitro* hormone-responsiveness: (i) generalised hormone independence; (ii) continued sensitivity to oestradiol and to the antioestrogenic activity of ICI 182780 but overall resistance to 4OHT; and (iii) stimulation by oestradiol and 4OHT and inhibition of both oestradiol- and 4OHT-stimulated growth by ICI 182780. Although the results of these primary culture experiments must be viewed as preliminary because of the small patient numbers involved, it is noteworthy that four previous studies have found evidence of stimulation by tamoxifen of breast cancer cells cultured directly from patients (Simon *et al.*, 1984*b*; Manni *et al.*, 1985; Osborne *et al.*, 1985; Nomura *et al.*, 1990).

It is concluded from this study that the Courtenay-Mills soft-agar clonogenic assay is suitable for investigating the effects of endocrine agents on the growth of established breast cancer cell lines and metastatic breast cancer cells taken directly from patients. At drug concentrations exceeding 10 nM, the novel steroidal antioestrogen, ICI 182780, exhibits a more complete and potent antioestrogenic effect than 4OHT on colony formation by hormone-responsive breast cancer cells and, unlike 4OHT, ICI 182780 exhibits no demonstrable agonist activity. Investigation of the effects of these agents in metastatic breast cancer cells taken from patients at the time of relapse on tamoxifen provides preliminary evidence to suggest that some patients may fail on tamoxifen therapy because of the oestrogenic activity of the parent drug and/or its metabolites and that treatment with a pure antioestrogen may be effective in patients with advanced breast cancer who appear to be resistant to tamoxifen.

**Abbreviations:** ER, oestrogen receptor; CFE, colony-forming efficiency; SCM, serum-containing growth medium; $SFM_{PR}$, serum-free growth medium containing phenol red; $SFM_{PRF}$, serum-free/phenol red-free growth medium; 4OHT, 4-hydroxytamoxifen; ICI 182780, 7α-[9-(4,4,5,5,5-pentafluoropentylsulphinyl)nonyl]oestra-1,3,5,(10)-triene-3, 17β-diol.

### References

BARDON, S., VIGNON, F., MONTCOURRIER, P. & ROCHEFORT, H. (1987). Steroid receptor-mediated cytotoxicity of an antiestrogen and an antiprogestin in breast cancer cells. *Cancer Res.*, **47**, 1441-1448.

BERTHOIS, Y., KATZENELLENBOGEN, J. & KATZENELLENBOGEN, B. (1986). Phenol red is in tissue culture media is a weak estrogen: implications concerning the study of estrogen-responsive cells in culture. *Proc. Natl Acad. Sci. USA*, **83**, 2496-2500.

CALVO, F., CARNEY, D., BRAVER, M. & MINNA, J. (1983). Hormone supplemented media for cloning human breast cancer: increased colony formation without alteration of chemosensitivity. *Br. J. Cancer*, **48**, 683-688.

CANNEY, P., GRIFFITHS, T., LATIEF, T. & PRIESTMAN, T. (1987). Clinical significance of tamoxifen withdrawal response. *Lancet*, **i**, 36.

COURTENAY, V. & MILLS, J. (1978). An *in vitro* colony assay for human tumours grown in immune-suppressed mice and treated *in vivo* with cytotoxic agents. *Br. J. Cancer*, **37**, 261-268.

DARBRE, P., YATES, J., CURTIS, S. & KING, R. (1983). Effect of estradiol on human breast cancer cells in culture. *Cancer Res.*, **43**, 349-354.

DARBRE, P., CURTIS, S. & KING, R. (1984). Effects of oestradiol and tamoxifen on human breast cancer cells in serum-free culture. *Cancer Res.*, **44**, 2790-2793.

DEVLEESCHOUWER, N., LEGROS, N., OLEA-SERRANO, N., PARIDAENS, R. & LECLERCQ, G. (1987). Estrogen conjugates and serum factors mediating the estrogenic trophic effect on MCF-7 cell growth. *Cancer Res.*, **47**, 5883-5887.

DITTRICH, C., JAKESZ, R., WRBA, F., HAVELEC, L., HAAS, O., SPONA, J., HOLZNER, H., KOLB, R. & MOSER, K. (1985). The human tumour cloning assay in the management of breast cancer patients. *Br. J. Cancer*, **52**, 197-203.

FAVONI, R., NOONAN, D. & DE CUPIS, A. (1993). Comparison between the anti-estrogenic activity of non-steroidal and steroidal compounds on human breast cancer cells *in vitro*. *Proc. Am. Soc. Cancer Res.*, 243.

GOLDENBERG, G. & FROESE, E. (1982). Drug and hormone sensitivity of estrogen receptor-positive and -negative human breast cancer cells *in vitro*. *Cancer Res.*, **42**, 5147-5151.

GOTTARDIS, M. & JORDAN, V. (1988). Development of tamoxifen-stimulated growth of MCF-7 tumors in athymic mice after long term antiestrogen administration. *Cancer Res.*, **48**, 5183-5187.

GREEN, M., WHYBOURNE, A., TAYLOR, I. & SUTHERLAND, R. (1981). Effects of antioestrogens on the growth and cell kinetics of cultured human mammary carcinoma cells. In *Non-steroidal Antioestrogens*, Sutherland, R. & Jordan, V.C. (eds) pp. 397-412. Academic Press: Sydney.

HAMBURGER, A. & SALMON, S. (1977). Primary bioassay of human tumor stem cells. *Science*, **197**, 461-463.

HENDERSON, I. (1991). Endocrine therapy of metastatic breast cancer. In *Breast Diseases*, Harris, J., Hellman, S., Henderson, I.C., Kinne, D.W. (eds) pp. 559-604. J.B. Lippincott: Philadelphia.

HOWELL, A., DODWELL, D., ANDERSON, H. & REDFORD, J. (1992). Response rate after withdrawal of tamoxifen and progestogens in advanced breast cancer. *Ann. Oncol.*, **3**, 611-617.

HUG, V., HAYNES, M., RASHID, R., SPITZER, G. & HORTOBAGGYI, G. (1984). Improved culture conditions for clonogenic growth of primary breast tumours. *Br. J. Cancer*, **50**, 207-213.

JONES, S., DEAN, J., YOUNG, L. & SALMON, S. (1985). The human tumor clonogenic assay in human breast cancer. *J. Clin. Oncol.*, **3**, 92-97.

KATZENELLENBOGEN, B., KENDRA, K., NORMAN, N. & BERTHOIS, Y. (1987). Proliferation, hormonal responsiveness, and estrogen receptor content of MCF-7 human breast cancer cells grown in the short-term and long-term absence of estrogens. *Cancer Res.*, **47**, 4355-4360.

KERN, D., CHIEN, F. & MORTON, D. (1984). Selective effects of insulin and hydrocortisone on colony formation and chemosensitivity of human tumours in soft agar. *Int. J. Cancer*, **33**, 807-812.

KODAMA, F., GREENE, G. & SALMON, S. (1985). Relation of estrogen receptor expression to clonal growth and antiestrogen effects on human breast cancer cells. *Cancer Res.*, **45**, 2720-2724.

LIPPMAN, M., BOLAN, G. & HUFF, K. (1976). The effects of estrogens and antiestrogens on hormone-responsive human breast cancer in long-term tissue culture. *Cancer Res.*, **36**, 4595-4601.

LIPPMAN, M., AITKEN, S. & ALLEGRA, J. (1981). Regulation of growth and DNA synthesis by oestrogens and antioestrogens in human breast cancer cell lines. In *Non-steroidal Antioestrogens*, Sutherland, R. & Jordan, V.C. (eds) pp. 365-395. Academic Press: Sydney.

MANNI, A., WRIGHT, C., PONTARI, M., FELL, P. & JOEHL, R. (1985). Hormone dependency of human breast neoplasms cultured *in vitro* in the stem cell assay. *J. Natl Cancer Inst.*, **74**, 767-770.

MANNI, A., WRIGHT, C. & BUCK, H. (1991). Growth factor involvement in the multihormonal regulation of MCF-7 breast cancer cell growth in soft agar. *Breast Cancer Res. Treat.*, **20**, 43-52.

NOMURA, Y., TASHIRO, H. & HISAMATSU, K. (1989). *In vitro* clonogenic growth and metastatic potential of human operable breast cancer. *Cancer Res.*, **49**, 5288-5293.

NOMURA, Y., TASHIRO, H. & HISAMATSU, K. (1990). Differential effects of estrogen and antiestrogen on *in vitro* clonogenic growth of human breast cancers in soft agar. *J. Natl Cancer Inst.*, **82**, 1146-1149.

OSBORNE, C., VON HOFF, D. & MULLINS, K. (1985). Endocrine therapy testing of human breast cancers in the soft agar clonogenic assay. *Breast Cancer Res. Treat.*, **6**, 229-235.

OTTESTAD, L., TVEIT, K., HØIFØDT, H., NESLAND, J., VAAGE, S., HØIE, J., LUND, E. & PIHL, A. (1988). Cultivation of human breast carcinoma in soft agar. Experience with 237 fresh tumour specimens. *Br. J. Cancer*, **58**, 8-12.

OTTESTAD, L., TVEIT, K., HANNISDAL, E., SKVEDE, M., NESLAND, J. & GUNDERSEN, S. (1989). Colony forming efficiency of human breast carcinomas: lack of prognostic significance. *Br. J. Cancer*, **60**, 216-219.

PATHAK, M., MATRISIAN, L., MAGUN, B. & SALMON, S. (1982). Effect of epidermal growth factors on clonogenic growth of primary human tumour cells. *Int. J. Cancer*, **30**, 745-750.

REDDEL, R. & SUTHERLAND, R. (1984). Tamoxifen stimulation of human breast cancer cell proliferation *in vitro*: a possible model for tamoxifen tumour flare. *Eur. J. Cancer Clin. Oncol.*, **20**, 1419-1424.

ROOS, W., HUBER, P., OEZE, L. & EPPENBERGER, U. (1982). Hormone dependency and the action of tamoxifen in human mammary carcinoma cells. *Anticancer Res.*, **2**, 157-162.

SIMON, W., HANSEL, M., DIFTEL, M., MATTHIESEN, I., ALBRECHT, M. & HOLZEL, F. (1984*a*). Alteration of steroid hormone sensitivity during the cultivation of mammary carcinoma cells. *In Vitro*, **20**, 157-166.

SIMON, W., ALBRECHT, M., TRAMS, G., DIETEL, M. & HOLZEL, F. (1984*b*). *In vitro* growth promotion of human mammary carcinoma cells by steroid hormones, tamoxifen and prolactin. *J. Natl Cancer Inst.*, **73**, 313-321.

SMALLWOOD, J., MORGAN, G., COOPER, A., KIRKHAM, N., WILLIAMS, C., WHITEHOUSE, J. & TAYLOR, I. (1984). Correlations between clonogenicity and prognostic factors in human breast cancer. *Br. J. Surg.*, **71**, 109-111.

STAQUET, M., BROWN, B., ROZENCWEIG, M., VAN MUYLDER, E., DODION, P. & SANDERS, C. (1987). Validation of the clinical predictive values of the *in vitro* phase II clonogenic assay in cancer of the breast and ovary. *Am. J. Clin. Oncol.*, **10**, 485-490.

STROBL, J. & LIPPMAN, M. (1979). Prolonged retention of estradiol by human breast cancer cells in tissue culture. *Cancer Res.*, **39**, 3319-3327.

THOMPSON, E., KATZ, D., SHIMA, T., WAKELING, A., LIPPMAN, M. & DICKSON, R. (1989). ICI 162384, a pure antagonist of estrogen-stimulated MCF-7 cell proliferation and invasiveness. *Cancer Res.*, **49**, 6929-6934.

TVEIT, K., ENDRESSEN, L., RUGSTAD, H., FODSTAD, O. & PIHL, H. (1981). Comparison of two soft-agar methods for assaying the chemosensitivity of human tumours *in vitro*: malignant melanoma. *Br. J. Cancer*, **51**, 843-852.

VIGNON, F., BOUTON, M. & ROCHEFORT, H. (1987). Antiestrogens inhibit the mitogenic effect of growth factors on breast cancer cells in the complete absence of estrogens. *Biochem. Biophys. Res. Commun.*, **146**, 1502-1508.

VON HOFF, D., CLARK, G., STOGDILL, B., SAROSDY, M., O'BRIEN, M., CASPER, J., MATTOX, D., PAGE, C., CRUZ, A. & SANDBACH, J. (1983). Prospective clinical trial of a human tumor cloning system. *Cancer Res.*, **43**, 1926-1931.

WAKELING, A., NEWBOULT, E. & PETERS, S. (1989). Effects of antioestrogens on the proliferation of MCF-7 human breast cancer cells. *J. Mol. Endocrinol.*, **2**, 1-10.

WAKELING, A., DUKES, M. & BOWLER, J. (1991). A potent specific pure antioestrogen with clinical potential. *Cancer Res.*, **51**, 3867-3873.

WELSHONS, W., WOLF, M., MURPHY, C. & JORDAN, V. (1988). Estrogenic activity of phenol red. *Mol. Cell Endocrinol.*, **57**, 169-178.

WILKS, D. & WEST, C.M.L. (1991). A serum-free medium for the Courtenay-Mills soft agar assay. *Int. J. Cell Cloning*, **9**, 559-569.

WOSIKOWSKI, K., KUNG, W., HASMANN, M., LOSER, R. & EPPENBERGER, U. (1993). Inhibition of growth-factor-activated proliferation by anti-estrogens and effects on early gene expression of MCF-7 cells. *Int. J. Cancer*, **53**, 290-297.